# Production of Terpenoids by Synthetic Biology Approaches

**DOI:** 10.3389/fbioe.2020.00347

**Published:** 2020-04-24

**Authors:** Caizhe Zhang, Kui Hong

**Affiliations:** Key Laboratory of Combinatorial Biosynthesis and Drug Discovery, Ministry of Education, School of Pharmaceutical Sciences, Wuhan University, Wuhan, China

**Keywords:** terpenoids, synthetic biology, metabolic engineering, *Escherichia coli*, *Saccharomyces cerevisiae*

## Abstract

Terpenoids are a large family of natural products with remarkable diverse biological functions, and have a wide range of applications as pharmaceuticals, flavors, pigments, and biofuels. Synthetic biology is presenting possibilities for sustainable and efficient production of high value-added terpenoids in engineered microbial cell factories, using *Escherichia coli* and *Saccharomyces cerevisiae* which are identified as well-known industrial workhorses. They also provide a promising alternative to produce non-native terpenes on account of available genetic tools in metabolic engineering and genome editing. In this review, we summarize the recent development in terpenoids production by synthetic biology approaches.

## Introduction

Terpenoids, also known as terpenes or isoprenoids, are a large family of natural products. More than 80,000 different terpenoids have been found in almost all life forms. Structural diversity of terpenoids makes them a wide range of applications as pharmaceutical, biofuels, and flavors. The skeletons of terpenoids are derived by condensation of multiple units of isopentenyl diphosphate (IPP) and its isomer dimethylallyl diphosphate (DMAPP), which are naturally generated by either mevalonate (MVA) pathway in eukaryotes and methylerythritol-phosphate (MEP) pathway in prokaryotes and plant plastids. In addition to these natural routes, synthetic routes for non-natural precursors have also been reported (Kang et al., 2016; Chatzivasileiou et al., 2019; Clomburg et al., 2019). Core structures of terpenes are then post-modified by cytochromes P450s (P450s) that play a vital role in endowing various bioactivities to terpenoids.

Production of terpenoids from natural resources may encounter technical challenges. For instance, ginsenoside Rh2, a potent candidate for cancer therapy, is a triterpenoid saponin derived from *Panax* species (Wong et al., 2015). Its content in dried *Panax* ginseng roots is less than 0.01% (Wang et al., 2019). Using the synthetic biology approach in engineered yeast, the yield of ginsenoside Rh2 has reached 2.25 g/L in fed-batch fermentation (Wang et al., 2019). This result provides an excellent case for improving cell factories to produce plant rare natural products. The rapid advances in synthetic biology suggest an alternative sustainable approach to achieve the industrial scale of terpenoids production (Bian et al., 2017; Clomburg et al., 2017; Wang et al., 2018). However, several significant challenges remain in microbial biosynthesis as a general approach for the supply of valuable terpenoids, including (i) the biological parts for genetic circuits construction have not been sufficiently characterized; (ii) the post-modifications of terpenoids remains inefficient; and (iii) the toxic accumulation of intermediate products and insufficient supply of precursors. Therefore, a platform that can provide available genetic tools and a comprehensive understanding of its metabolism is urgently needed. In this purpose, *Escherichia coli* and *Saccharomyces cerevisiae* have been used as ideal platform hosts for various creative explorations (Table 1). In this review, we focus on recently developed strategies specific to address challenges that in the pathway efficiencies optimization, gene circuits construction and regulation, pathway programing, subcellular engineering and co-culture strategy of terpenoids biosynthesis using synthetic biology approaches.

**TABLE 1:** Strategies for production of various terpenoids in *S. cerevisiae* and *E. coli*

Product	Strategy and features	Culture conditions	Titer or Improvement	References
***S. cerevisiae***				
8-hydroxygeraniol	Mitochondrial compartmentalization by targeting the geraniol biosynthetic pathway to the mitochondria	Fed-batch fermentation	227 mg/L	Yee et al., 2019
Geraniol	Protein structure analysis, site-directed mutation, overexpression of tHMGR and IDI	Fed-batch fermentation	1.68 g/L	Jiang et al., 2017
Limonene	Regulation of ERG20 by P_HXT__1_ promoter (glucose-sensitive)	Fed-batch fermentation	917.7 mg/L 6-fold	Cheng et al., 2019
	N-degron-mediated destabilization of ERG20	Batch fermentation	76 mg/L	Peng et al., 2018
Amorpha-4,11-diene	Optimization of [NADPH]/[NADP^+^] ratios by introducing mutations into phosphofructokinase (PFK) along with overexpression of ZWF1	Shake flasks	497 mg/L	Kwak et al., 2019
	Mitochondria compartmentalization by targeting the whole FPP pathway together with Amorpha-4,11-diene synthase (ADS) into mitochondria	Shake flasks	427 mg/L	Yuan and Ching, 2016
Zerumbone	Regulation of ERG9 by P_HXT__1_ promoter	Fed-batch fermentation	40 mg/L	Zhang et al., 2018a
Farnesene	Increase the availability of acetyl-CoA by removing the native source of cytosolic acetyl-CoA (Δ*RHR2*) and overexpressing xPK, PTA, ADA and NADH-HMGr	Fed-batch fermentation	2.24 g/L/h >130 g/L	Meadows et al., 2016
Oxygenated taxanes	*E. coli–S. cerevisiae* co-culture by dividing the synthetic pathway for the acetylated diol paclitaxel precursor into two modules	Co-culture in bioreactor	33 mg/L	Zhou et al., 2015
Nerolidol	Minimizing competition for FPP by destabilizing squalene synthase, degrade ER membrane-integrating protein.	Two-phase flask	4–5.5 g/L	Peng et al., 2017
Casbene	Regulation of ERG20 and ERG9 by P*_HXT__1_* and P*_*ERG*__1_* promoters	Deepwell microplate	108.5 mg/L	Callari et al., 2018
Jolkinol C	Optimize soluble expression of Cbsp using protein tagging strategies, codon-optimization of CYPs	Milliliter plates	800 mg/L	Wong et al., 2018
Carotenoid	Colorimetric-based promoter strength comparison system; inducer/repressor-free sequential control strategy by combining a modified GAL regulation system and a P_HXT__1_-controlled squalene synthetic pathway	Fed-batch fermentation	1156 mg/L	Xie et al., 2015
Lycopene	Lipid engineering; Improve triacylglycerol metabolism	Fed-batch fermentation	2.37 g/L	Ma et al., 2019
	Scaffold-free enzyme assemblies (IDI and CrtE);	Fed-batch fermentation	2.3 g/L	Kang W. et al., 2019
Medicagenic acid	Endoplasmic reticulum (ER) engineering; expand the ER by disrupting the phosphatidic acid phosphatase	Tube cultures	27.1 mg/L 6-fold	Arendt et al., 2017
β-Carotene	Tri-functional CRISPR system combines transcriptional activation, transcriptional interference, and gene deletion	Tube cultures	2.8-fold	Lian et al., 2017
Squalene	ER engineering; expand the ER by overexpressing a key ER size regulatory factor, INO2.	Shake flasks	634 mg/L	Kim J.E. et al., 2019
***E. coli***				
Total monoterpenoids	Non-natural route to isoprenoid biosynthesis (isoprenoid alcohol pathway/IPA)	Shake flasks	0.6 g/L	Clomburg et al., 2019
Pinene	Adaptive laboratory evolution for improving pinene tolerance; E. coli co-culture system; whole-cell biocatalysis	Shake flasks	166.5 mg/L	Niu et al., 2018
	Cell-free enzyme systems for production of monoterpenes from glucose	Glass vials	14.9 g/L	Korman et al., 2017
Limonene	Cell-free enzyme systems	Glass vials	12.5 g/L	Korman et al., 2017
Geranyl acetate	Two-phase system; convert monoterpenoid geraniol to its acetyl ester to avoid geraniol toxicity	Fed-batch fermentation	4.8 g/L	Chacón et al., 2019
Viridiflorol	Promoters and RBSs engineering	Fed-batch fermentation	25.7 g/L	Shukal et al., 2019
α-Bisabolol	CRISPRi-guided balancing of MVA pathway	Shake flasks	25 mg/L	Kim et al., 2016
	FPP-resistant mevalonate kinase 1; lower MVA pathway; Optimization of inducer concentration, aeration and enzymatic cofactor	Fed-batch fermentation	8.5 g/L	Kim S. et al., 2019
Oxygenated taxanes	Modular engineering (MEP, cyclase, and P450 module), promoters engineering	Fed-batch fermentation	570 mg/L	Biggs et al., 2016
Longifolene	Codon optimization of longifolene synthase, investigate into different FPP synthases	Fed-batch fermentation	382 mg/L	Cao et al., 2019
Ophiobolin F	Ophiobolin synthase with SUMO tag; phylogenetics based mutation	Shake flasks	150.5 mg/L	Yuan et al., 2019
Carotenoids	Scaffold-free enzyme assemblies (IDI and CrtE)	Fed-batch fermentation	276.3 mg/L 5.7-fold	Kang A. et al., 2019
Astaxanthin	Promoters and RBSs engineering; multidimensional heuristic process (MHP)	Fed-batch fermentation	320 mg/L	Zhang et al., 2018b
	CRISPR-mediated morphology and oxidative stress engineering	Shake flasks	11.92 mg/g DCW	Lu and Liu, 2019
Zeaxanthin	Dynamic control of MVA pathway by IPP/FPP-responsive promoter.	Fed-batch fermentation	722.46 mg/L	Shen et al., 2016
Lycopene	CRISPRi-guided balancing of MVA pathway	Shake flasks	71.4 mg/L	Kim et al., 2016
	Optimization of the lycopene biosynthetic; Overexpressing the MEP pathway	Shake flasks	448 mg/g DCW	Coussement et al., 2017

## Genetic Circuits and Dynamic Control

As the basic genetic elements of biosynthetic pathways, biological parts (e.g., promoter, terminator, ribosome-binding site (RBS), regulatory protein, etc.) should be well-characterized and optimized for synthetic biology. Constitutive or inducible promoters with different strengths are always the major theme in synthetic biology. Their efficiency are also affected by the combination of terminators and RBS.

Comparing to constitutive promoters, inducible promoters possess a strong capacity to start gene expression only under specific culture conditions. For example, the glucose-sensing promoter *HXT1*(P*_*HXT*__1_*) is strong at high glucose concentrations and weak at low glucose concentrations. Using glucose-responsive promoters also avoids the need for expensive repressors or inducers (Scalcinati et al., 2012; Xie et al., 2015; Zhao et al., 2017; Cheng et al., 2019). By using P*_*HXT*__1_*, the competing gene farnesyl diphosphate synthase (ERG20) for the consumption of precursors IPP and DMAPP was inhibited, and the carbon flux was reallocated from the growth pathway to the limonene synthetic pathway, and the limonene titer reached 917.7 mg/L in fed-batch fermentation (Cheng et al., 2019). When each of MVA pathway enzymes was transcribed from high-expression galactose-regulated promoters (*P_*GAL*__1_* or *P_*GAL*__10_*), an amorpha-4,11-diene yield of more than 40 g/L was resulted (Westfall et al., 2012). FPP (farnesyl diphosphate) is the intermediate of MVA pathway, but exhibits toxicity when it accumulates in *E. coli* (Martin et al., 2003). Whole-genome transcript arrays identified an FPP-responsive promoter answering to the accumulation of FPP (Dahl et al., 2013). Using IPP/FPP-responsive promoter in *E. coli*, Shen et al. (2016) coordinated the expression of all genes of the MVA pathway from *S. cerevisiae* using the tunable intergenic regions to increase the availability of FPP. The dynamically regulated MVA pathway prevented the toxic accumulation of IPP/FPP, and the titer of zeaxanthin reached 722.46 mg/L in fed-batch fermentation (Supplementary Figure S1A). P*_*ERG*__1_* represents an ergosterol-sensitive promoter, was shown to restrict squalene synthase (ERG9) expression levels efficiently (Yuan and Ching, 2015). Callari et al. (2018) replaced the promoter regions of *ERG20* and *ERG9* with P*_*HXT*__1_* and P*_*ERG*__1_* to redirect the flux from FPP and sterols, generated a titer of 108.5 mg/L of casbene.

Clustered regularly interspaced short palindromic repeats interference (CRISPRi) uses a catalytically-inactive Cas9 protein (dCas9) and a single guide RNA (gRNA) to repress the expression of targeted genes by blocking transcription (Qi et al., 2013). Kim et al. (2016) established a dynamic regulation CRISPRi system to coordinate the metabolic flux between cell growth and IPP/DMAPP accumulation. An L-rhamnose-inducible promoter was used to control the expression of dCas9. During the production phase, L-rhamnose was removed to restore gene expression, and the production of lycopene and α-bisabolol increased. Lian et al. (2017) developed a CRISPR-AID system using three orthogonal CRISPR proteins combines. When *HMG1* was overexpressed, down-regulation of *ERG9* and deletion of *ROX1* could significantly increase the production of β-carotene in *S. cerevisiae*. These genes were chosen as the targets for CRISPRa (transcriptional activation), CRISPRi, and CRISPRd (gene deletion), respectively (Supplementary Figure S1B).

The modular pathway engineering group multiple genes into modules to reduce regulatory complexities and help to unlock the potential of the multi-gene pathway for the production of terpenoid products (Ajikumar et al., 2010; Supplementary Figure S1C). Keasling’s group tuned the expression of multiple genes within operons by generating libraries of tunable intergenic regions and balancing the expression of MVA pathway, which resulted in a 7-fold increase in mevalonate production (Pfleger et al., 2006). Zhang et al. (2018b) reported a multidimensional heuristic process for astaxanthin production. Astaxanthin biosynthesis pathway was grouped into four modules, that each module controlled by different promoter of pre-determined strength, and get a yield of 320 mg/L in *E. coli*. Through screening of combinations of promoters and terminators, valencene synthase expression cassette was optimized to reach a titer of 539.3 mg/L (Chen et al., 2019). When Shukal et al. (2019) introduced viridiflorol synthase (VS) from *Agrocybe aegerita* to *E. coli*, three T7 promoter variants were characterized for different pathway expression, and RBS libraries that covered a broad range of translational initiation rates were optimized. The yield of viridiflorol was increased to 25.7 g/L in fed-batch fermentation.

## Pathway Enzyme Design

Directed evolution and rational protein design have been used to engineer enzymes in heterologous pathways (Eriksen et al., 2014; Niu et al., 2018; Hong et al., 2019; Supplementary Figure S1E). Monoterpenes are synthesized from geranyl diphosphate (GPP), which is also the precursor for the biosynthesis of FPP. Therefore, preventing the consumption of GPP by restricting FPP formation is profitable to produce monoterpenes. Mendez-Perez et al. (2017) introduced a mutation (Ser81 to Phe) in native FPP synthase of *E. coli*, resulting in an enzyme that preferentially synthesizes GPP instead of FPP, and the engineered strains yielded 653 mg/L of 1,8-cineole and 505 mg/L of linalool, which are 30- and 5-fold improvement, respectively. Jiang et al. (2017) demonstrated that two essential amino acid residues Y436 and D501 located in active pocket of the key enzyme geraniol synthase are critical for the key step of dephosphorylation. By overexpression of truncated 3-hydroxy-3-methylglutaryl-coenzyme reductase (tHMGR) and isopentenyl diphosphate isomerase (IDI), the highest titer of 1.68 g/L geraniol was achieved in fed-batch fermentation in *S. cerevisiae.*

Protein tagging strategies are effective means for enzyme engineering. A truncated gene encoding casebene synthase from *Jatropha curcas* with various protein tags was integrated into a geranylgeranyl diphosphate (GGPP)-producing strain, which yields 160 mg/L of casbene (Wong et al., 2018). Using a small ubiquitin-like modifier (SUMO) fusion tag and phylogenetics based mutations, ophiobolin synthase solubility and activity were improved. The yield of sesterterpene ophiobolin F was increased to 150.51 mg/L in *E. coli* (Yuan et al., 2019). Endoplasmic reticulum-associated protein degradation decreased cellular levels of ERG9, and increased the titer of sesquiterpene nerolidol to 100 mg/L (Peng et al., 2017). Also, N-degron-mediated destabilization of ERG20 improved the production of monoterpenes of 18 mg/L linalool or 76 mg/L limonene in *S. cerevisiae* (Peng et al., 2018). To decrease the concentration of pivotal enzyme, a synthetic degradation has been established based on *Mesoplasma florum* tmRNA system (Cameron and Collins, 2014). Based on the CRISPRi and the N-end rule for protein stability, Martínez et al. (2017) described a genome editing approach by changing the rates of both RNA synthesis and protein degradation. Synthetic protein scaffolds provide precise control of metabolic flux by preventing the loss of intermediates to diffusion or competing pathways (Dueber et al., 2009). However, scaffolded enzyme assemblies have different limitations, as enzymes fused in large structures may encounter a decrease or complete loss of the activity (Jia et al., 2014). Recently, Kang W. et al. (2019) developed a scaffold-free modular enzyme assembly by employing a pair of short peptide tags. The GGPP synthase and IDI were modularly assembled, which increased carotenoid production by 5.7-folds in *E. coli* and yielded a titer of 2.3 g/L lycopene in *S. cerevisiae*.

## Reprograming and Design New Precursor Biosynthetic Pathways

High intracellular levels of the essential intermediate IPP, may cause growth inhibition, reduce cell viability and plasmid instability (Martin et al., 2003; George et al., 2014, 2018). To explore more efficient and practical terpenoids biosynthetic pathways, non-natural pathways were developed. Kang et al. reported an alternative IPP-bypass MVA pathway by utilizing promiscuous activities of phosphomevalonate decarboxylase and an *E. coli* endogenous phosphatase, which successfully decoupled isopentenol production from IPP generation, and remarkably improved isoprenol titer to 3.7 g/L in batch cultures (Kang et al., 2016; Kang A. et al., 2019). Clomburg et al. (2019) constructed an isoprenoid alcohol pathway (IPA) for terpenoids synthesis, which could convert isoprenoid precursors through a minimal number of steps, and less ATP consumption. Chatzivasileiou et al. (2019) established an isopentenol utilization pathway (IUP) for bioconversion of isopentenols, isoprenol, or prenol to IPP or DMAPP. The IUP is composed of choline kinase (from *S. cerevisiae*), isopentenyl phosphate kinase, and isopentenyl-pyrophosphate delta isomerase and requires ATP as its sole co-factor, whereas much more straightforward than the current MVA or MEP alternatives.

Acetyl-CoA is also the critical branch-point precursor for terpenoids biosynthesis. However, in *S. cerevisiae*, acetyl-CoA is compartmentalized that mainly derived from pyruvate in mitochondria and fatty acids degradation in the peroxisome (Hammer and Avalos, 2017). Meadows et al. (2016) rewired the central carbon metabolism of *S. cerevisiae* to improve redox balance and enable biosynthesis of cytosolic acetyl-CoA with a reduced ATP requirement. The engineered strains produced 25% more farnesene while requiring 75% less oxygen, and sustaining stable yield for 2 weeks that reaches >130 g/L farnesene. This system has provided a reference for all terpenoids and other acetyl-CoA-derived compounds. Lu et al. (2019) constructed a synthetic acetyl-CoA pathway, in which, the catalytic activity of glycolaldehyde synthase was improved by directed evolution. Then the acetyl-phosphate synthase was selected based on the phylogenetic tree of PKs, which converts glycolaldehyde into acetyl-phosphate (AcP). AcP could be used to generate acetyl-CoA by the phosphate acetyltransferase. It is the shortest pathway from formaldehyde to acetyl-CoA.

In *S. cerevisiae*, NADPH production highly depends on the oxidative pentose phosphate pathway (Minard and McAlister-Henn, 2005). Kwak et al. (2019) engineered mutated phosphofructokinase (PFK) along with overexpression of glucose-6-phosphate dehydrogenase to reduce glycolytic metabolic fluxes, resulted in substantial increases of [NADPH]/[NADP^+^] ratios. Moreover, amorpha-4,11-diene was overproduced in *S. cerevisiae* achieved a titer of 497 mg/L with a 3.7-fold increase compared to the parental strain.

## Subcellular Engineering and Cell Free System

**FIGURE 1:**
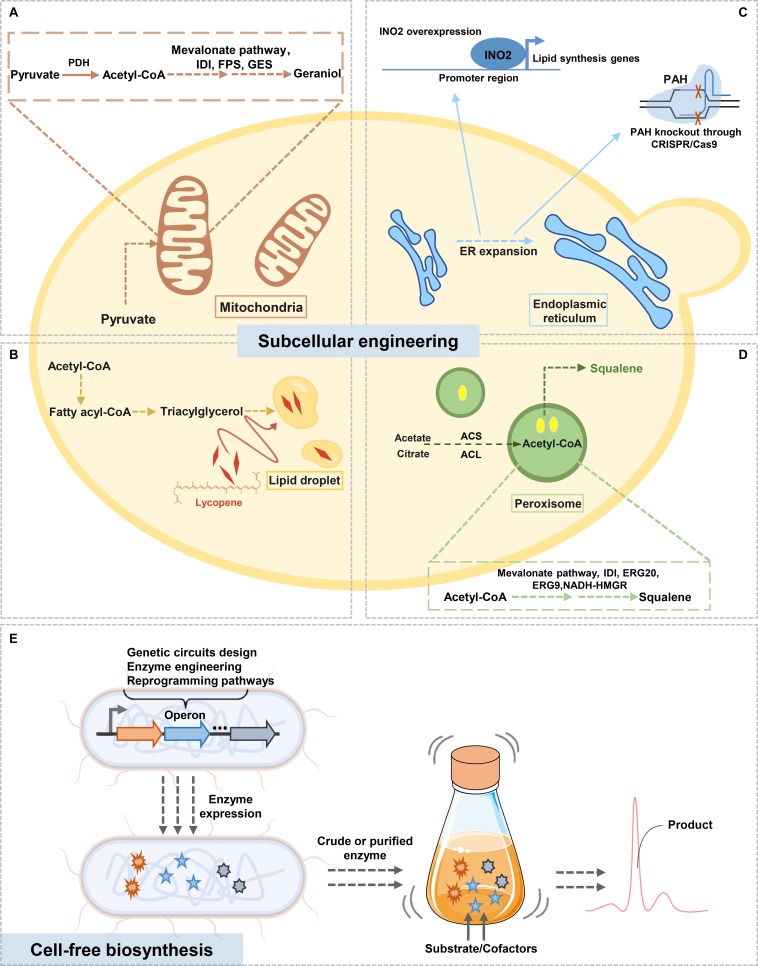
Overview of terpenoids biosynthesis by subcellular engineering **(A–D)**, and cell-free terpenoids biosynthesis **(E)**. Yeast cells contain various subcellular organelles (e.g., mitochondria, endoplasmic reticulum, etc.) which provide beneficial circumstances for different terpenoids biosynthetic pathways. Cell-free biosynthesis systems by *in vitro* reconstructing the entire biosynthetic pathway is another efficient solution for terpenoid production. PDH, pyruvate dehydrogenase; IDI, isopentenyl diphosphate isomerase; FPS, farnesyl diphosphate synthase; GES, geraniol synthase; PAH, phosphatidic acid phosphatase; ACS, acetyl-CoA synthetase; ACL, ATP-dependent citrate lyase; ERG20, farnesyl diphosphate synthase; ERG9, squalene synthase; NADH-HMGR, NADH-specific HMG-CoA reductase.

Compared with cytosol, mitochondria provide a compartmentalized environment with higher reducing redox potential. There is a growing interest in utilizing the acetyl-CoA pool in mitochondria for the biosynthesis of value-added compounds. By transplanting the whole FPP pathway together with amorpha-4,11-diene synthase into yeast mitochondria, the yield of amorpha-4,11-diene in engineered strain reached 427 mg/L (Yuan and Ching, 2016). Yee et al. (2019) targeted the geraniol biosynthetic pathway to the *S. cerevisiae* mitochondria to protect the GPP pool from consumption by the cytosolic ergosterol pathway. The production of geraniol in mitochondrial was 6-fold increase compared to cytosolic producing strains (Figure 1A). Lipid droplets (LDs) are ubiquitous organelles that store metabolic energy in the form of neutral lipids. Ma et al. (2019) established a lipophilic lycopene production strategy in *S. cerevisiae* by using LDs accumulation. A non-oleaginous *S. cerevisiae* for triacylglycerols production was combined with their composition adjustment and LDs size regulation. Therefore, lycopene accumulated continuously to 2.37 g/L in 5 days (Figure 1B). Expansion of the endoplasmic reticulum (ER) could increase yeast metabolic capacity. Arendt et al. (2017) reported that the disruption of the phosphatidic acid phosphatase (PAH) resulted in the expansion of the ER, which stimulated the production of triterpene biosynthesis enzymes and increased triterpenoid and triterpene saponin accumulation. Kim J.E. et al. (2019) engineered *S. cerevisiae* to expand the ER by overexpressing a key ER size regulatory factor, INO2. The production of squalene was increased by 71-fold, with the titer of 634 mg/L in shake flask fermentation (Figure 1C). Liu et al. (2020) compartmentalized yeast peroxisome as a subcellular factory for squalene biosynthesis. Hybridization of the cytoplasm- and peroxisome-engineered strains was constructed, and squalene with a titer of 11.0 g/L was reached in two-stage fed-batch fermentation (Figure 1D).

On the other hand, cell free biosynthesis (CFB) systems are easy to use multiple enzyme pathways sourced from various organisms, and also overcome the challenges of precursor supply and products toxicity. The purified enzyme system and crude cell extract system are common application forms of CFB systems (Dudley et al., 2015; Li et al., 2018; Figure 1E). CFB systems designed by Korman et al. (2017) converted glucose into monoterpenes and can be self-sustaining for long periods. The platform contains 27 enzymes and using glycolysis reconstituted to generates both ATP, NADPH, and acetyl-CoA, resulting in the production of 12.5 g/L limonene and 14.9 g/L pinene. In addition, CFB systems provide great flexibility for biochemical pathways study. Chen et al. (2017) utilized *in vitro* metabolic engineering to reveal the regulatory network of a reconstituted amorpha-4,11-diene synthetic pathway, and identified the inhibition of ATP on both FPP synthase and amorpha-4,11-diene synthase.

## Cytochromes P450 and Post-Modifications of Terpenoids

Cytochromes P450 (P450s) play a crucial role in yielding final terpenoid products with wide chemical diversity and bioactivities. *S. cerevisiae* is a favored host for expressing P450s on account of advanced protein expression mechanism, abundant intracellular membranes and the inherent benefits of large-scale microbial fermentation (Paddon et al., 2013; Gold et al., 2018; Wong et al., 2018; Zhang et al., 2018a). It is generally deemed that the ability to express soluble P450s in *E. coli* is limited. The main challenge is the lack of an endomembrane system for attachment of the eukaryotic P450s, as they have a helical hydrophobic transmembrane domain containing 20–30 amino acid residues at their N-terminal ends. Transmembrane domain truncation and N-terminal replacement are vastly used for heterologous expression of eukaryotic P450s. Ichinose and Wariishi (2013) performed extensive heterologous expression of fungal P450s in *E. coli* using 304 of P450 isoforms and identified N-terminal amino acid sequences that can significantly improve chimeric P450s expression levels. They revealed that the choice of combinations of N-terminal and catalytic domains is critical for high-level expression. Biggs et al. (2016) demonstrated *E. coli* could be a feasible host for P450-mediated terpenoid biosynthesis. In their study, the first module, “MEP,” was comprised of the rate-limiting enzymes of the IPP-producing MEP pathway. The second “cyclase” module was comprised of taxadiene synthase (TxS) and GGPP synthase. The relatively low expression of a five-copy plasmid with a weak promoter was essential for P450 and CPR functionality. Besides, with reductase partner interactions and N-terminal modifications, a record yield of 570 mg/L of oxygenated taxanes was achieved in *E. coli*.

The important post-modifications of terpenoids also include hydroxylation by P450s and glycosylation by glycosyltransferases. Gold et al. (2018) showed a platform for the production of steviol glucosides (SGs) in *S. cerevisiae*. Two P450s of kaurene oxidase (KO) and kaurenoic acid hydroxylase (KAH) are required in succession in the conversion of kaurene into steviol. By optimizing the copy number modulation of KO-KAH-CPR combinations, the conversion was maximized. Wang et al. (2015, 2019) established a series of cell factories to produce ginsenoside Rh2 by optimizing UDP-glycosyltransferase bioparts expression. Combined with precursor (protopanaxadiol) supply optimization, the titer of ginsenoside Rh2 reached 2.25 g/L in fed-batch fermentation.

## Co-Culture of Engineered Strains

A newly approach of co-culture engineering to enhance terpenoids production was developed. In some conditions, a single host cell cannot provide an optimal environment for functioning all pathway enzymes, and metabolic burdens from overexpression of complex pathways may reduce biosynthetic efficiency (Zhang et al., 2015; Wang et al., 2020). By dividing the acetylated diol paclitaxel precursor synthetic route into two modules, expressed in either *S. cerevisiae* or *E. coli*, a stable co-culture was achieved in the bioreactor. The engineered *E. coli* strain accomplishes the biosynthesis of the intermediate taxadiene. Meanwhile, *S. cerevisiae* is the preferred host for cytochrome P450 (P450s) expression, using this two-component system, oxygenated taxanes with a titer of 33 mg/L was overproduced (Zhou et al., 2015; Supplementary Figure 1D). Similarly, Niu et al. (2018) constructed an *E. coli* - *E. coli* co-culture system for pinene biosynthesis. The MEV pathway and heterologous pathway (the GPP synthase and pinene synthase) were engineered in different pinene tolerance *E. coli* strains, respectively. The optimization of whole-cell biocatalysis, which could separate cell growth and production phase, improved pinene production to 166.5 mg/L.

## Conclusion

Over the last few decades, biological engineers achieved grand developments in synthetic biology. The enormous potential of *E. coli* and *S. cerevisiae* as platform strains has been confirmed with various successes. However, as synthetic biology targets are progressively more complicated, there remain some challenges to engineering industrial hosts because of the lack of knowledge of complex biochemical and cellular metabolism and its regulation. With the fast development of synthetic biology tools such as CRISPR-Cas9, adaptive laboratory evolution (ALE) combine with next-generation sequencing and high-throughput screening, it promises to reach a deeper understanding of cellular metabolism. The capacity of DNA synthesis has been made great progressed over the past decade, and it is conventional to synthesize the large gene cluster for terpenoids biosynthesis. In addition, new DNA assembly methods facilitate the speedy construction of different genetic part combinations or to replace genetic parts in a single step. Besides, dynamic control, compartmentalization, module design, or cell-free system are practical methods to enhance the overall reaction efficiency of multi-enzyme pathways.

## Author Contributions

Both authors conceived the review, wrote and reviewed the manuscript.

## Conflict of Interest

The authors declare that the research was conducted in the absence of any commercial or financial relationships that could be construed as a potential conflict of interest.
