# Supplementary figures and images for: Production of Terpenoids by Synthetic Biology Approaches
Source: Front Bioeng Biotechnol. 2020 Apr 24;8:347. doi: 10.3389/fbioe.2020.00347 (PMC7193017; doi:10.3389/fbioe.2020.00347)

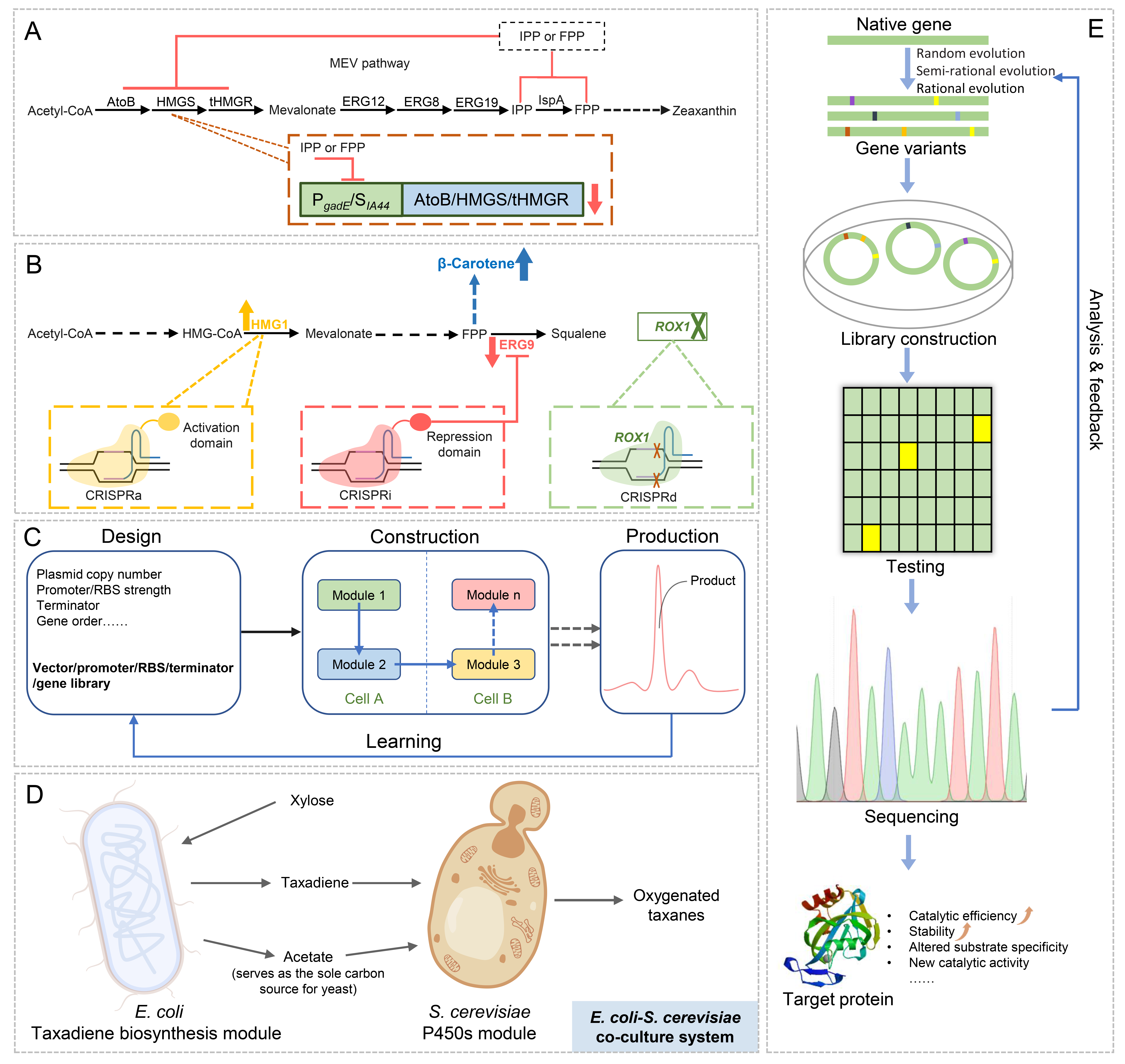

Supplement: Supplementary file 1 [file Image_1.tif]
