# Supplementary material for: Production of Terpenoids by Synthetic Biology Approaches
Source: Front Bioeng Biotechnol. 2020 Apr 24;8:347. doi: 10.3389/fbioe.2020.00347 (PMC7193017; doi:10.3389/fbioe.2020.00347)
Supplement: Supplementary file 2 [file Table_1.docx]

**Supplementary Figure 1** | Overview of genetic circuits design (**A**, **B**, **C**), co-culture of engineered strains (**D**), and strategies for protein evolution (**E**). AtoB, acetyl-CoA acetyltransferase; HMGS, HMG-CoA synthase; tHMGR, truncated HMG-CoA reductase; ERG12, mevalonate kinase; ERG8, phosphomevalonate kinase; ERG19, mevalonate pyrophosphate decarboxylase; IspA, farnesyl diphosphate synthase; *S_IA44_*, sensor IA44; HMG1, HMG-CoA reductase; ERG9, squalene synthase; *ROX1*, encoding a stress responsive transcriptional regulator;
